# Perceived neighborhood environment, social capital and life satisfaction among older adults in Shanghai, China

**DOI:** 10.1038/s41598-022-10742-7

**Published:** 2022-04-23

**Authors:** Nan Lu, Bei Wu

**Affiliations:** 1 Department of Social Work and Social Policy, School of Sociology and Population Studies, Renmin University of China, Beijing, China; 2 Sau Po Centre on Ageing, The University of Hong Kong, Pok Fu Lam, Hong Kong SAR China; 3 Rory Meyers College of Nursing and NYU Aging Incubator, New York University, 433 First Avenue, New York, NY 10010 USA

**Keywords:** Geriatrics, Quality of life

## Abstract

This study examined the mediator role of social capital on the association between perceived neighborhood environment and life satisfaction among older adults in urban China, and further tested the moderating effect of gender in the above paths (i.e., from neighborhood environment to life satisfaction; from neighborhood environment to social capital; from social capital to life satisfaction). We used quota sampling approach to recruit 472 respondents aged 60 years old or older in Shanghai in 2020. From the perspective of structural equation modeling, multiple group analysis was conducted to examine the proposed hypotheses. The measurement model of social capital was well established in urban Chinese community contexts. Based on the whole sample, the results of the mediation model showed that social capital played a mediation role in the association between neighborhood environment and life satisfaction. Furthermore, the results of multiple group analysis showed that the association between neighborhood environment and cognitive social capital was only significant among older women. The findings highlight the role of neighborhood environment and social capital in building age-friendly communities.

## Introduction

The global population aging process has accelerated rapidly, and this trend will continue in the next few decades. China has the largest older population in the world^1^. According to the recent 7th national census of China, the Chinese population aged 60 years and older reached 264 million in 2020, which accounts for 18.7% of the total Chinese population. On one hand, given the increased life expectancy, the issue of sustaining and promoting both physical and subjective well-being in later life is a major task for policy makers, researchers, and health care providers in China. On the other hand, the average Chinese family size has decreased from 4.41 in 1982 to 2.62 in 2020^1^. Moreover, intergenerational geographic distances have widened due to the prominent phenomenon of migrant populations. The number of migrant populations reached 492 million in 2020. Combined with the influences of urbanization and modernization, these factors led to significant transitions in the traditional family-based aging care system in China^2^. Under such circumstances, community-based services and supports could play a more salient role in sustaining well-being among older populations in the next few decades.

Life satisfaction is frequently used to measure the cognitive dimension of subjective well-being in later life and therefore, is recognized as a crucial indicator of healthy aging^3^. Older adults who are satisfied with their lives are more likely to remain not only independently functional but also actively involved in social activities to pursue personal and collective interests. Policy and interventions around subjective well-being in later life therefore, should pay attention to modifiable and culturally sensitive factors of life satisfaction in later life.

Previous literature has identified factors associated with life satisfaction, including socioeconomic status, physical health, living alone, interpersonal relationships, and access to health care^4,5^. Furthermore, neighborhood environment was found to play an important role in promoting life satisfaction and other mental well-being indicators in later life^6–9^.

Neighborhood environment is a multi-dimensional concept and includes physical neighborhood environment and social neighborhood environment^10,11^. While the former includes transportation, outdoor spaces and buildings, and community services, the later includes social participation, volunteering, trust, and reciprocity^7,10,12–14^. In particular, community-based social capital (hereafter social capital), an important social supportive resource embedded from individuals’ social connections, is recognized as a key indicator of social neighborhood environment. Social capital could sustain the well-being of older adults through promoting social support, information diffusion of health knowledge, health service utilization, and health behavior^8,15^.

As compared with objective physical neighborhood environment indicators, older adults’ perceived physical neighborhood environment was found to be a stronger factor of their mental well-being^16,17^. This might because that physical neighborhood environment represents the availability of supportive resources in the neighborhood, whereas perceived neighborhood environment reflects variations in individuals’ access and their utilization levels to such resources. Therefore, in this research, we used older adults’ subjective evaluations to assess physical neighborhood environment.

In summary, perceived neighborhood environment and social capital were found to be two crucial factors related to life satisfaction among older adults^8,18,19^. A few empirical studies have been conducted to examine neighborhood environment and well-being among older adults in Chinese context^20–23^. However, few studies have used comprehensive instruments to measure social neighborhood environment (e.g., social capital, which will be discussed in later sections). There is also lack of studies examining how neighborhood environment affects social capital, what the pathways that link these two factors with life satisfaction, and what factors that potential moderate these associations in the context of developing countries and regions. Therefore, this study measured social capital from both cognitive and structural dimensions, tested the mediation effects of social capital on the relationship between neighborhood environment and life satisfaction and further examined the moderator role of gender in these associations in urban Chinese community contexts.

As the most economically developed city in China, Shanghai aims to develop age friendly communities and promote the quality of neighborhood environment in all local rural and urban communities by 2035. For example, the city plans to develop community health service centers and day care centers that deliver regular physical examinations, need assessments, primary care, prevention, long-term care, and hospice care for older residents in all communities. Walkable environments and the accessibility and safety of transportation are other development goals. Moreover, Shanghai also plans to develop safe, livable environment, educational programs preventing fraudulent activities, and neighborhood watch for older adults living alone and those with disabilities. Furthermore, it plans to develop community organizations and activities to promote older adults’ participation in community activities, peer support groups, and volunteering. Older adults are also encouraged to participate in community development and administration. However, these programs are in the process of being developed, and it is important to provide updated evidence on whether these activities have an impact on well-being among older adults.

### Neighborhood environment and life satisfaction

According to ecological model of ageing, both physical and social neighborhood environments play important roles in promoting well-being in later life through person-environment interactions^6,7,24,25^. For older adults, good-quality physical neighborhood environment (e.g., adequate access to health care, safe living environment, and good transportation conditions) can provide resources and access that facilitate them to be more adaptive to their age-related changes in social connections and health status, which in turn, may further enhance their satisfaction levels with their lives^26–28^. For example, older adults’ physical health and capacities of mobility tend to decline over time. In this case, availability of health care services in the communities and access to local transportation may play a crucial role in helping older adults to meet their medical and daily care needs. Furthermore, neighborhood safety and good access to transportation could help older adults to sustain social participation, fulfil social roles, and enhance their service utilization levels^9,29^. Additionally, these factors can be modified and enhanced with appropriate attention and resources. Therefore, we focused on these neighbourhood indicators in this study.

Proponents of ecological model of ageing also put great emphasis on the potential influence of physical environment on social environment, and their effects on health outcomes in later life^30^. For example, high-quality of physical neighborhood environment (e.g. accessible public transportation and safe living environment) could lead to the increase of social capital (e.g., high levels of sense of belonging to local community, active participation in community activities and volunteering)^9,31–33^. Therefore, we argue that physical neighborhood environment could affect life satisfaction of older adults through community-based social capital.

The majority of research on neighborhood environment and life satisfaction among older adults has been conducted in developed and Western countries and regions^7,10,11^. Although the literature supports the role of neighborhood environment in enhancing life satisfaction in later life, findings on the associations between specific neighborhood environment indicators and life satisfaction are mixed^6,19,34–36^. For example, transportation, community support, health services, and social environment were found to be significantly associated with life satisfaction among Canadian older adults^36^. Park and Lee^6^ found that perceived neighborhood environment indicators such as transportation, social services, social participation, and social inclusion were significantly associated with life satisfaction among older adults in Seoul, South Korea. In a recent study conducted in Hong Kong, transportation and social participation were associated with life satisfaction^35^. However, community health services were not associated with life satisfaction among adults aged 75 years or older^35^. Based on a nationally representative sample, Xie^19^ found that older adults’ perception of local amenities was significantly associated with life satisfaction among older adults in urban China. However, the association between community services and life satisfaction was nonsignificant. Regarding community safety, studies revealed that the perceived neighborhood safety was positively associated with life satisfaction^37^; while neighborhood crime was negatively related to older adults’ quality of life^38^.

In summary, the majority of studies showed that transportation and community safety were significantly associated with life satisfaction in later life. Mixed results were identified in research concerning the relationship between community health care and life satisfaction. Presumably, these mixed results might partially result from the lack of consensus on the conceptualization and measurement of social and physical aspects of the neighborhood environment^7,24^. Different measurements have been used in empirical studies, making the comparison of the findings even more difficult. Furthermore, there is a lack of examination of the interplay between physical neighborhood environment and social capital, especially in developing country contexts.

### The mediating role of social capital

The conceptualization and operationalization of social capital can be examined from multiple perspectives (e.g., collectivist and individualist) and levels (e.g., micro and macro)^3,39^. As a commonly used definition of social capital in the health-related field, Putnam conceptualized social capital from a collectivist perspective and described it as “features of social organization, such as trust, norms, and networks, that can improve the efficiency of society by facilitating coordinated actions”^40^. Social capital also refers to individuals’ supportive resources based on their social connections in local communities, where people share cultural values and social norms and organization memberships^41^. Furthermore, social capital can be measured at both individual and group levels (e.g. community and country levels by aggregation of individual data)^39^. In this study, social capital was measured at individual level to reflect individual differences in access to and benefits from community-based social supportive resources.

In this research, we considered individual-level social capital as a multidimensional concept, which can be measured by cognitive and structural dimensions^3^. Cognitive social capital is reflected by subjective appraisals of social trust and reciprocity in the community. Structural social capital, on the other hand, refers to objective aspects of social capital and is often measured by memberships in community organizations, volunteering, social participation in community activities, and citizenship activities to address common problems in the community^39^. Community social capital can not only provide older adults with social support and a social network, but also promote collective action in communities and enhance a sense of belonging and meaning in life, which is important in older age^3,8^.

Research on social capital and well-being (including life satisfaction) in later life has been mainly conducted in developed and Western contexts^8^. Systematic reviews found that individual-level social capital was a stronger factor linked to life satisfaction than collective social capital (e.g., community and country levels)^3,8^. Furthermore, the association between cognitive social capital and life satisfaction was found to be stronger than that between structural social capital and life satisfaction^3,8^.

Whereas social capital was recognized as an important factor of life satisfaction, research findings on the relationships between specific dimensions of social capital (i.e., cognitive and structural dimensions) and life satisfaction were inconclusive. For example, both cognitive and structural social capital were found to be significant factors related to life satisfaction among older adults in developed countries^8,42,43^. Specifically, trust, reciprocity, organization memberships, and citizenship activities were significantly associated with life satisfaction^42,44,45^. However, findings on the associations between country-level social capital indicators such as trust, volunteering, and organization memberships and life satisfaction were mixed in developed countries^46,47^. Furthermore, the association between structural social capital indicators and life satisfaction was found to be nonsignificant in Chinese contexts^48,49^.

Furthermore, low social trust and reciprocity were found in poor neighborhood with high crime prevalence rates^32^. The literature has found significant association between the perception of neighborhood characteristics and cognitive social capital indicators (e.g., a sense of communities)^31,33^. Perceived community safety was found to be associated with social trust and support among local residents^32^. Transportation was also found to be associated with sense of community in later life^33^. In addition, neighborhood resources, including transportation, was found to be associated with social participation (i.e., an indicator of structural social capital) among older adults living in Europe^50^. Safety and transportation were found to be related to the level of physical activities among residents^9^. The availability of elder care services and health services were significantly related to sense of community among older adults in Beijing and Hong Kong^20,51^. While the neighborhood environment refers to access to amenities and services in local communities, social capital can be used to diffuse health-related information, promote social participation, and enhance service utilization rates. In this case, the neighborhood environment provides the foundation for social capital to develop. We hypothesized that social capital would play a significant mediator role in the association between neighborhood environment and life satisfaction (Hypothesis 1).

### The moderating role of gender

There are limited numbers of studies which have examined gender differences in neighborhood environment, social capital, and life satisfaction, and that in the relationship between social capital and life satisfaction among older adults^18,52,53^. For example, previous research identified gender differences in neighborhood walking^52^. Compared with older men, older women tend to have higher levels of involvement in informal reciprocity among neighbors and friends, caring activities, and other activities held by community organizations. In contrast, older men are more likely to be involved in relatively formal social activities (e.g., local residents working together to solve common problems in the communities). Regarding the relationship between social capital and life satisfaction, although older women tend to benefit more from trust and reciprocity among neighbors^18,53^, older men benefit more from formal activities such as citizenship activities^18^.

Furthermore, there is a lack of research on the potential moderator role of gender in the influence of neighborhood environment on social capital and life satisfaction among older adults, especially for those living in the contexts of developing countries and regions. We argue that older men and women tend to have different social roles, and different preferences and expectations from social activities that they engage in. In this case, the neighborhood environment might have different effects on older women and men’s life satisfaction. Therefore, we proposed that gender would play a moderator role in the pathways linking neighborhood environment, social capital, and life satisfaction (Hypothesis 2).

## Methods

### Sampling

Data were based on a social survey titled “Social Capital, Intergenerational Solidarity, and Mental Health among Older Chinese Adults” (SCIENCE). Quota sampling was used to select respondents from Siping Street, Yangpu District, Shanghai, China, in summer 2020. As the most economically developed city in China, Shanghai has seen the aging process of its local population in Shanghai accelerate rapidly—around 35% of residents were aged 60 years or older in 2019 (the national average is 18.1%). Yangpu is in the northeast of Shanghai’s central area. The local economy of Yangpu district is at the medium level among the 16 districts in Shanghai. Siping Street consists of 23 communities. Tongji University is in this area. If the university staff and students were not counted, the proportion of residents aged 60 years or older is above 40%. Furthermore, Shanghai has initiated a second round of pilot projects to build age friendly communities for older adults with cognitive impairments. Siping Street is one of the participating streets in these projects. Therefore, this area is suitable for studying neighborhood environment, social capital and life satisfaction among older adults.

The sampling procedures were as follows: In the first step, we included all 23 communities from Siping Street. In the second step, we recruited 20 respondents from each community, with the support of local community centers. We controlled for the age and gender ratios of the respondents in each community to ensure consistency with those from a representative sample (collected in 2019) surveyed by the Shanghai Municipal Health Commission (e.g., age group 60–64: 48% men and 52% women; age group 75 or older: 41% men and 59% women). The inclusion criteria of the sample were: (a) aged 60 years or older; (b) had local household registration status; (c) lived in local communities for more than 6 months in the past 12 months; and (d) passed a cognitive screening test based on the Short Portable Mental Status Questionnaire^54^. The cutoff point was 6 for those with a high school education or lower and 7 for those with a college education or higher^54^.

Six trained interviewers conducted face-to-face interviews with respondents at community centers. For those with limited functional health, interviews were conducted at their home. The survey included rich information in terms of older adults’ sociodemographic characteristics, depressive symptoms, loneliness, life satisfaction, neighborhood environment, and social capital. Of 498 respondents, 476 agreed to participate in the survey and successfully completed the interview. Four respondents did not pass the cognitive screening test. The response rate was 94.77%. Ethics approval was obtained from the University Ethics Committee. All methods were performed in accordance with the relevant guidelines and regulations. Informed consent forms were obtained before collecting the data. The final sample size was 472.

### Measurement

#### Life satisfaction variable

Life satisfaction was measured by the 8-item Life Satisfaction Scale-Chinese, which was designed to measure life satisfaction among older Chinese populations^55^. The respondents were asked about the degree of their satisfaction with eight life domains: friendship, family ties, spouse relationship, family interactions, health, food, financial status, and housing. Responses were measured on a 5-point Likert scale (0 = strongly disagree, 2 = neutral, 4 = strongly agree). We calculated mean values to represent life satisfaction levels (Cronbach’s alpha = 0.837).

#### Social capital variable

In this research, social capital was assessed by two latent constructs, namely cognitive social capital and structural social capital^56^. Each latent construct has four indicators (details were shown in data analysis section). We selected the eight indicators based on the social capital scale from the World Bank and the recommendations of previous systematic reviews^39,57^. Specifically, four statements were used to assess cognitive social capital: (a) The majority of residents living in the local community can be trusted; (b) Most residents provide support to each other; (c) The residents care about both their and others’ interests; and (d) The local community gives me a feeling of family and I have a sense of belonging to this family. Responses were measured on a 5-point Likert scale (0 = strongly disagree, 2 = neutral, 4 = strongly agree).

The other four indicators were used to assess structural social capital. The number of organization memberships for each respondent in the past year was calculated based on the following list: political organizations, religious groups, work unions, women’s groups, community associations, sports groups, charity groups, professional associations, neighborhood committees, and community colleges for older adults. The frequency of the respondents’ participation in social activities organized by these social organizations in the past year were assessed based on a 6-point Likert scale, ranging from 1 (never) to 3 (several times per year) to 6 (twice or more per week). The respondents were also asked whether they worked with other residents to solve common problems in local communities in the last year (0 = never, 2 = occasionally, 4 = almost always) and whether they engaged in volunteering activity in the past 30 days (0 = no, 1 = yes).

#### Perceived neighborhood environment variable

Instead of objective measure of neighborhood, perceived indicators of neighborhood environment were found to be a stronger factor linked to mental well-being^16^; thus, neighborhood environment was measured by older residents’ subjective appraisals in this study. The respondents were asked about their general satisfaction levels with three domains of the neighborhood environment: community health care (e.g., community health service centers, day care centers, and hospitals), community safety (e.g., quality of security services, crime rates), and transportation conditions (e.g., public transport and traffic condition). Responses were measured on a 5-point Likert scale (0 = very unsatisfied, 2 = fair, 4 = very satisfied).

#### Moderator and covariates

The moderator was gender, assessed by a binary variable (0 = male, 1 = female). Regarding covariates, age was measured in years. Marital status, living arrangement, and educational attainment were assessed by binary variables (1 = married, 0 = other; 1 = living alone, 0 = living with others; 1 = secondary school education or higher, 0 = primary school education or lower). The log value of household income per year was calculated. Furthermore, the respondents were asked whether they have any of 14 doctor-diagnosed chronic diseases, including diabetes, arthritis, hypertension, stroke, and cardiovascular diseases. Summed scores were calculated to represent the number of chronic diseases. Finally, cognitive function was assessed by Short Portable Mental Status Questionnaire^54^, with a range of 0–10. Higher scores indicate higher cognitive function.

### Data analysis

We used structural equation modeling (SEM) in to examine the mediation model of perceived neighborhood environment, social capital, and life satisfaction. From a SEM perspective, a multiple-group analysis was further conducted to test the gender difference in the mediation model. Mplus 7.0 was used^56^. This approach, widely used in empirical studies, allowed us to test whether gender moderates the mediation model^58,59^.

The analytic procedures were as follows: First, we conducted a measurement model of cognitive and structural social capital using confirmatory factor analysis. The following fit indexes and cutoff points were used to determine whether the model adequately fit the data: the chi-square test statistic (nonsignificant estimate indicates good model fit), weighted root mean square residual (WRMR; less than 1 indicates good fit); Tucker–Lewis index (TLI; > 0.90 indicates good fit), comparative fit index (CFI; > 0.90 indicates good fit), root mean square error of approximation (RMSEA; < 0.05 indicates good fit), and standardized root mean square residual (SRMR; < 0.08 indicates good fit)^60^.

Second, we conducted a structural model to regress life satisfaction on perceived neighborhood environment while controlling covariates. Furthermore, structural and cognitive social capital were entered in the structural model to test the indirect effect of perceived neighborhood environment on life satisfaction through social capital.

Finally, we built multiple-group models to test the moderation role of gender in the mediation model. The specific procedures were as follows: First, we tested the measurement model in each gender group separately. Second, factor loadings of latent variables were held equal between the two gender groups. This allowed us to conduct meaningful comparisons between the groups^61^. Third, outcome variable and covariates were added to the structural model. We tested the moderation effects of gender on the relationships among neighborhood environment, social capital, and life satisfaction using Wald tests. All missingness in key variables was less than 3%. Listwise deletion was used in the analysis.

### Ethics approval

Ethics approval was obtained from the Ethics Committee of the University of Hong Kong in April 2020 (EA200113).

## Results

### Sample characteristics

Table 1 shows the sociodemographic characteristics of older respondents. The respondents’ average age was 68.37 years, 56.4% were women, more than 80% were married, and only 12.7% lived alone at the time of the survey. Around half of the respondents reported their monthly household income was 9,000 RMB or less, and 57.8% completed a high school education or more. The average number of chronic diseases was 1.37. The most common chronic diseases were hypertension (47.5%), heart disease (18.4%), diabetes or high blood sugar (16.3%), and arthritis (9.7%). On average, around 75% of the respondents were satisfied or very satisfied with their lives (average score of 4 or higher).

**Table 1:** Sample characteristics (*N* = 472).

	*n* (%)	*M* (*SD*)
**Age**		68.37 (6.69)
60–74	378 (80.1)	
75 or older	93 (19.7)	
**Gender**		
Women	266 (56.4)	
Men	206 (43.6)	
**Marital status**		
Married	392(83.1)	
Other status	79 (16.7)	
**Lived alone**	60 (12.7)	
**Household income**		
0–9,000RMB per month	216 (45.8)	
9,001 RMB or more per month	243 (51.5)	
**Education level**		
Secondary school education or lower	199 (42.2)	
High school education or higher	273 (57.8)	
**Cognitive Function**		9.40 (0.88)
**Number of diseases**		1.37 (1.41)
**Satisfaction with health care service**		4.24 (0.63)
**Satisfaction with transportation**		4.43 (0.54)
**Satisfaction with community safety**		4.32 (0.59)
**Social trust**		4.22 (0.67)
**Helpfulness of other residents**		4.29 (0.57)
**Willingness to collaborate with others**		4.20 (0.61)
**A sense of belonging**		4.34 (0.52)
**Organization memberships**		2.58 (1.23)
**Social participation**		3.50 (1.34)
**Citizenship activities**		2.10 (1.25)
**Volunteering**		0.65 (0.48)
**Life satisfaction**		4.27 (0.48)

100 RMB = $15.52 USD.

### The mediation model

We established a measurement model of cognitive and structural social capital first. Fit index estimates showed a good model fit: χ^2^(19) = 27.824, *p* = 0.0869, RMSEA = 0.031, CFI = 0.990, TLI = 0.985, WRMR = 0.563. Regarding cognitive social capital, the factor loading standardized estimates were between 0.615 and 0.927. Regarding structural social capital, the standardized estimates were between 0.621 and 0.873.

Moreover, the first structural model was conducted by regressing life satisfaction on neighborhood environment, χ^2^(9) = 4.731, *p* = 0.8571, RMSEA = 0.000, CFI = 1.000, TLI = 1.020, SRMR = 0.012 (See Table 2). Community health care, safety, and transportation conditions were all significantly associated with life satisfaction (health care: *b* = 0.182, *SD* = 0.037, *p* < 0.001; transportation: *b* = 0.153, *SD* = 0.049, *p* < 0.01; safety: *b* = 0.200, *SD* = 0.045, *p* < 0.001).

**Table 2:** Statistical models for life satisfaction among older adults.

	Structural model 1			Structural model 2		
	*b*	*SE*	β	*b*	*SE*	β
Age	0.003	0.003	.049	0.007	0.003	.097*
gender	0.114	0.035	.119**	0.109	0.040	.113**
Marital status	0.088	0.088	.068	0.069	0.100	.053
Educational attainment	0.032	0.036	.033*	0.008	0.036	.009
Living arrangement	0.070	0.086	.049	0.092	0.071	.064
Household income	0.169	0.050	.157**	0.161	0.043	.149***
Number of chronic diseases	− 0.066	0.013	− .194***	− 0.065	0.013	− .194***
Cognitive function	− 0.035	0.020	− .063	− 0.031	0.024	− .057
Community health care	0.182	0.037	.241***	0.152	0.022	.200***
Transportation condition	0.153	0.049	.173**	0.131	0.037	.147***
Community safety	0.200	0.045	.246***	0.122	0.027	.149***
Cognitive social capital				0.281	0.045	.255***
Structural social capital				0.037	0.023	.069

*p < .05; **p < .01; ***p < .001.

The second structural model was conducted by entering social capital variables, χ^2^(104) = 122.735, *p* = 0.1013, RMSEA = 0.020, CFI = 0.989, TLI = 0.984, WRMR = 0.635 (See Table 2). This model explained 49.0% of the variance in life satisfaction. Cognitive social capital was significantly associated with life satisfaction, whereas the association between structural social capital and life satisfaction was nonsignificant (cognitive: *b* = 0.281, *SD* = 0.045, *p* < 0.001; structural: *b* = 0.037, *SD* = 0.023, *p* = 0.098). The associations between health care, safety, transportation and life satisfaction all remained significant (health care: *b* = 0.152, *SD* = 0.022, *p* < 0.001; transportation: *b* = 0.131, *SD* = 0.037, *p* < 0.001; safety: *b* = 0.122, *SD* = 0.027, *p* < 0.001). Furthermore, health care was associated with cognitive social capital, but not with structural social capital (cognitive: *b* = 0.112, *SD* = 0.032, *p* < 0.001; structural: *b* = − 0.028, *SD* = 0.089, *p* = 0.756). Transportation was not associated with social capital (cognitive: *b* = 0.067, *SD* = 0.040, *p* = 0.094; structural: *b* = 0.059, *SD* = 0.132, *p* = 0.655). Safety was associated with both social capital latent variables (cognitive: *b* = 0.250, *SD* = 0.031, *p* < 0.001; structural: *b* = 0.231, *SD* = 0.099, *p* < 0.05).

The mediating role of cognitive social capital in the relationship between community health care, community safety, and life satisfaction was statistically significant (health care and life satisfaction: *b* = 0.031, *SD* = 0.010, *p* < 0.01; safety and life satisfaction: *b* = 0.070, *SD* = 0.014, *p* < 0.001). In other words, approximately 17% and 35% of the effect of health care and community safety on life satisfaction were mediated by cognitive social capital, respectively. The mediating role of cognitive social capital in the relationship between transportation and life satisfaction was not significant (*b* = 0.019, *SD* = 0.012, *p* = 0.115). The mediating role of structural social capital in the relationships between the neighborhood environment and life satisfaction was nonsignificant (health care and life satisfaction: *b* = − 0.001, *SD* = 0.003, *p* = 0.762; transportation and life satisfaction: *b* = 0.002, *SD* = 0.005, *p* = 0.659; safety and life satisfaction: *b* = 0.009, *SD* = 0.007, *p* = 0.193).

### Multiple-group analysis

We further examined gender differences in the relationships between neighborhood environment, cognitive social capital, and life satisfaction. Because the association of structural social capital and life satisfaction was nonsignificant for both men and women, structural social capital was not included in the multiple-group analysis.

First, measurement models of cognitive social capital were established in both gender groups, even after factor loadings of cognitive social capital were held equal between the two gender groups, χ^2^(6) = 6.167, *p* = 0.4048, RMSEA = 0.011, CFI = 1.000, TLI = 1.000, WRMR = 0.049. In other words, we established factor loading invariance in this latent variable.

Second, life satisfaction, neighborhood environment, and covariates were entered in the final model, χ^2^(73) = 82.887, *p* = 0.2008, RMSEA = 0.024, CFI = 0.995, TLI = 0.991, SRMR = 0.047. Cognitive social capital was significantly associated with life satisfaction among both women and men (women: *b* = 0.259, *SD* = 0.090, *p* < 0.01; men: *b* = 0.329, *SD* = 0.077, *p* < 0.001). The moderation effect of gender on the relationship between cognitive social capital and life satisfaction was statistically nonsignificant, Wald χ^2^(1) = 0.363, *p* = 0.5469. Furthermore, gender differences in the relationship between the three neighborhood environment indicators and life satisfaction and those between community safety, transportation, and cognitive social capital were nonsignificant.

**Figure 1:**
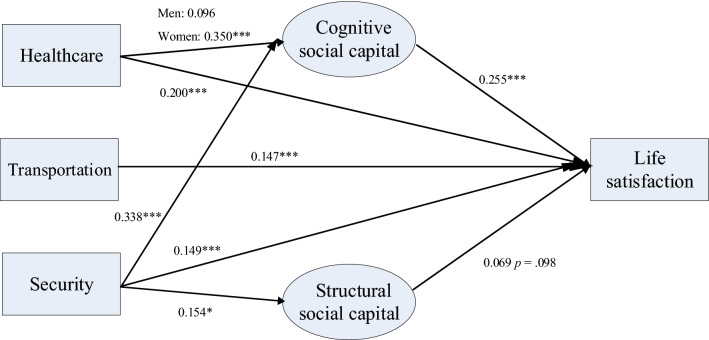
The Structural Model of Perceived Neighborhood Environment, Social Capital and Life Satisfaction: The Moderator Role of Gender. *Notes:* Only standardized coefficients are reported. *p < .05 (two-tailed); ***p < .001 (two-tailed); The covariates were controlled in the model.

However, the results identified a significant moderating effect of gender on the relationship between community health care and cognitive social capital, even when all of the structural paths from neighborhood environment and cognitive social capital to life satisfaction were held equal across the two gender groups [χ^2^(83) = 91.666, *p* = 0.2414, RMSEA = 0.021, CFI = 0.996, TLI = 0.993, SRMR = 0.076; Wald chi-square (1) = 5.152, p < 0.05]. In comparison to men, community health care had a stronger association with life satisfaction among women (women: β = 0.350, *SD* = 0.078, *p* < 0.001; men: β = 0.096, *SD* = 0.081, *p* = 0.236). The mediator role of cognitive social capital in the association between community health care and life satisfaction varied by gender. This mediation was only statistically significant among women (women: *b* = 0.065, *SD* = 0.019, *p* < 0.01; men: *b* = 0.018, *SD* = 0.015, *p* = 0.252; see Fig. 1).

## Discussion

The present study aimed to test a mediation model of neighborhood environment, social capital, and life satisfaction among older adults living in urban China, as well as the moderator role of gender in the paths of the above mediation model. The findings represent new contributions by highlighting the role of social capital in the relationship between neighborhood environment and life satisfaction, and also further examining gender differences in these associations. This study also adopted social capital theory to examine the influence of physical environment on social environment in the framework of the ecological model of aging.

Consistent with the findings of previous research, cognitive social capital, rather than structural social capital, was significantly related to life satisfaction when controlling for neighborhood environment. Furthermore, perceived neighborhood environment was significantly associated with life satisfaction in the final model. This finding is also consistent with those of previous studies. It provides further evidence on the importance of neighborhood environment and its impact on life satisfaction. Furthermore, cognitive social capital played a partial mediation role in the relationship between community health care, safety, and life satisfaction. It is likely that a good-quality neighborhood environment in terms of health care and safety could play an important role in fostering cognitive social capital (i.e., trust and reciprocity). Transportation, in contrast, tended to have a direct effect on life satisfaction, and did not influence social capital. Furthermore, social capital might enhance life satisfaction by enhancing supportive resources, sharing information, promoting health-related behavior, and promoting use of home- and community-based services. Finally, the findings add new empirical evidence by identifying gender differences in the relationship between community health care service and cognitive social capital. The above relationship was significant among women but not men. In Chinese culture, women are more likely to take on caregiving roles (e.g., care for sick family members and those with chronic conditions) than men. Good-quality of community health care services might provide opportunities for older women to not only facilitate information sharing among neighbors (e.g., attend health education programs organized by neighborhood committees and community hospitals; and share health knowledge and skills with neighbors), but also enhance informal reciprocity in the neighborhood (e.g., collaboration on caregiving tasks). In other words, community health care services are likely to affect life satisfaction among older women both directly and indirectly. For men, health care services are more likely to be a crucial source of health support, rather than important opportunities for socializing.

The findings have the following policy and intervention implications. First, both neighborhood environment and social capital should be included in older adults’ needs assessment and used to identify older adults who are at risk of poor life satisfaction and subjective well-being. Second, social capital policies and interventions should focus on not only promoting social trust and reciprocity in the neighborhood, but also helping older adults utilize available community- and home-based health services and outdoor public spaces. In doing so, the resources embedded in the neighborhood environment could be used in a more efficient manner, which could further promote a sense of life satisfaction. Third, older women’ participation in health care services could also be important opportunities for them to socialize. This could be used to enhance both health service utilization and social capital (e.g., social participation and reciprocity). Fourth, the development of neighborhood environment should emphasize older residents’ feelings of bonding and belonging to local communities. Both subjective and objective indicators should be used as part of the outcome evaluation of the neighborhood environment development projects.

### Limitations

The limitations of the study are as follows. First, given that the data were cross-sectional, we could not examine the causality between neighborhood environment, social capital, and life satisfaction. Furthermore, since perceived neighborhood environment, cognitive social capital, and life satisfaction were subjective and self-reported measures in this study, these factors might be affected by unobserved variables such as physical health conditions and personality traits. This issue could generate same-source bias. We controlled number of chronic diseases and cognitive function in the model. Future studies should further test the mediating effects of community-based social capital in the relationship between neighborhood environment and life satisfaction among older adults living in different social and economic context. Second, objective indicators of physical environment and community services such as walkable environment, and the availability of community elder care services were not included in this study. Moreover, in this study, we used three indicators, rather than latent construct to assess neighborhood environment. This allows us to test the gender difference in each pathway from neighborhood environment indicators to social capital and life satisfaction. Future studies should use a more comprehensive measurement of both physical and social environments of neighborhood environment, and further develop latent constructs of sub-dimensions of neighborhood environment in both rural and urban Chinese settings. Furthermore, given that we did not have access to the full list of residents living in Siping Street, we did not use random sampling to collect the data. Quota sampling was used to recruit the respondents. Although we matched the age and gender ratio of the sample to the population of the street, the sample may not be representative of the population of the area and the city of Shanghai.

In general, the study respondents reported relatively better physical health and higher income levels than national average levels. Future studies with a larger sample are needed to further this line of research.

## Conclusion

In conclusion, the findings show that perceived neighborhood environment not only directly affected life satisfaction, but also indirectly affected life satisfaction through community-based cognitive social capital. The findings also show that the relationship between health care services in the community and cognitive social capital was significant among older women but not older men. On one hand, the social and physical dimensions of neighborhood environment matter for life satisfaction. On the other hand, in-depth understanding of the pathway linking these factors to life satisfaction is needed to comprehensively evaluate the impact of neighborhood environment on subjective well-being among older adults.

## Data Availability

Data are available upon reasonable request.
